# Prescription patterns and therapeutic gaps among persons with epilepsy in Southwestern Nigeria

**DOI:** 10.3389/fphar.2024.1430716

**Published:** 2024-08-07

**Authors:** Luqman Ogunjimi, Bamidele Osalusi, Ayotomiwa Fagbemi, Ibironke Oyenuga, Fedora Ojini, Samuel Collins, Oluwatosin Elegbede, Olayinka Oladele, Fatai Fehintola

**Affiliations:** ^1^ Department of Pharmacology and Therapeutics, Obafemi Awolowo College of Health Sciences, Olabisi Onabanjo University, Remo Campus, Ago Iwoye, Nigeria; ^2^ Department of Medicine, Obafemi Awolowo College of Health Sciences, Olabisi Onabanjo University, Remo Campus, Ago Iwoye, Nigeria; ^3^ Department of Medicine, Federal Medical Centre Abeokuta, Abeokuta, Nigeria; ^4^ Department of Biochemistry, Obafemi Awolowo College of Health Sciences, Olabisi Onabanjo University, Remo Campus, Ago Iwoye, Nigeria; ^5^ Stroke Investigative and Research Network Unit, Department of Medicine, University College Hospital, Ibadan, Nigeria; ^6^ Department of Pharmacology and Therapeutics, University of Ibadan, Ibadan, Nigeria

**Keywords:** antiseizure medication, epilepsy, treatment gap, carbamazepine, polytherapy

## Abstract

**Introduction:** Pharmacotherapy with antiseizure medications (ASMs) has been a cornerstone for achieving long-term remissions in persons with epilepsy (PWEs). This study aims to determine the prescription patterns and treatment gaps (TGs) among PWEs.

**Methods:** Accordingly, a descriptive cross-sectional study was conducted with 940 PWEs aged ≥18 years having clinically confirmed diagnosis of epilepsy based on the International League Against Epilepsy (ILAE) criteria. At a scheduled interview with each participant, a previously established questionnaire was used to obtain clinical information relating to epilepsy in terms of the age of onset, etiology, duration of epilepsy, frequency, types, and number of ASMs used.

**Results:** There were fewer male participants [445 (47.4%) vs. 495 (53.6%)] than females, with a higher mean age of onset [(35.19 ± 21.10 vs. 31.58 ± 20.82 years; p = 0.009]. The medication characteristics showed that 336 (35.7%) of the 940 PWEs recruited were not on any ASMs, whereas the remaining 604 (64.3%) patients were on ASMs, with 504 (83.4%) on monotherapy vs. 100 (16.6%) on polytherapy. The PWEs on ASM monotherapy had a higher mean age [40.92 ± 19.40 vs. 33.61 ± 16.51 years; *p* < 0.001] and higher mean age of onset [34.47 ± 21.80 vs. 25.39 ± 19.78 years; *p* < 0.001] than those on polytherapy. Furthermore, there were more persons on ASM monotherapy among the participants with seizure duration < 2 years [251 (87.5%) vs. 36 (12.5%)] and seizure duration > 2 years [253 (79.8%) vs 64 (20.2%)].

**Conclusion:** The majority of the participants receiving ASMs were on monotherapy, with carbamazepine being the most frequently prescribed medication. Furthermore, about a third of the participants had TGs; therefore, healthcare providers should focus on alleviating the TGs among PWEs.

## 1 Introduction

Epilepsy is a chronic non-communicable disease that affects over 50 million people worldwide, with significant comorbidities affecting the quality of life as well as social, emotional, and economic statuses. There are increasing instances of lifetime burden and active epilepsy in developed countries, with prevalences of 6.8 million and 5.7 million, respectively (Thijs et al., 2019). Epilepsy is a significant public health problem in sub-Saharan African (SSA) countries, along with its therapeutic and diagnostic challenges. The use of antiseizure medications (ASMs) is one of the critical steps in the management of seizures. Many ASMs work through multiple mechanisms, which include presynaptic and postsynaptic blockade of the axonal Na channels, enhancement of inhibitory mechanisms, inhibition of excitatory mechanisms of transmission, Ca^+^ channel blockage, SV2A receptor blockage, and gamma-aminobutyric acid (GABA) modulation. ASMs that work by blocking the Na^+^-dependent action potentials include phenytoin, carbamazepine (CBZ), lamotrigine, topiramate, and zonisamide (Verrotti et al., 2020a). ASMs that work by modulating GABA include tiagabine, valproate, and vigabatrin. Although ASMs remain one of the bedrocks of seizure management, they are associated with various therapeutic and diagnostic limitations, such as lack of personnel, unavailability of diagnostic tools, poorly coordinated primary and secondary healthcare services, and rising burden of unawareness and understanding of the disease. However, the problems associated with availability, affordability, and tolerability of ASMs remain major challenges and contribute significantly to the increasing treatment gaps (TGs) in SSA countries. In a study to determine the TGs with respect to epilepsy in developing countries, Nwani et al. (2013) found a TG of 76% among persons with epilepsy (PWEs). These TGs mostly comprised people who were never diagnosed (36%) and those who were diagnosed but had poor drug adherence (38%). Non-adherence has been associated with high complication factors like lifetime epilepsy, increased risk of death, and increasing cost of healthcare among PWEs. In another study conducted in Niger, it was shown that among 60 patients, CBZ (75.6%), phenobarbital (24.2%), and both CBZ and phenobarbital (3.2%) were used as common medications (Assadeck et al., 2019). The present study aims to determine the prescription patterns and TGs among PWEs.

## 2 Methods

### 2.1 Study settings and design

A retrospective study was conducted among PWEs attending the Federal Medical Centre Abeokuta (FMCA), University College Hospital (UCH) of Ibadan, and Olabisi Onabanjo University Teaching Hospital (OOUTH) of Sagamu. The study was carried out after obtaining the due ethical approvals from all three study sites.

### 2.2 Diagnostic criteria and case ascertainment

A total of 940 PWEs aged 18 years and above who had a clinical history of seizures and no history of pseudoseizures or head trauma were included in the study. After the initial clinical diagnosis of epilepsy, the seizure classifications and descriptions of electroencephalography (EEG) findings were provided by two different neurologists based on the International League Against Epilepsy (ILAE) and Clinical Neurophysiology Society’s Standardized Critical Care EEG Terminology. In addition, the EEG signals were accurately recorded using the international 10–20 electrode standard placement from all participants using a Phoenix digital 6-channel EEG machine. All participants also preferably underwent brain magnetic resonance imaging (MRI) or cranial computed tomography (CT), which aided in the etiology of the epilepsy.

### 2.3 Patient evaluations

An interviewer-administered questionnaire containing a list of specific, direct, and definite preestablished questions was used to obtain the sociodemographic and clinical information related to epilepsy in terms of age, gender, level of education, age of onset of epilepsy, etiology, duration of epilepsy, frequency, and types of ASMs used. The study participants were fully informed of the research protocols detailing the purpose, methods, and benefits of the research, and consent forms duly signed by either the participants or guardians were obtained. None of the participants withdrew from the study.

### 2.4 Statistical analyses

The data were coded and analyzed using the IBM Statistical Package of Social Sciences version 23. The sociodemographic and clinical characteristics of the PWEs were presented as frequency (percentage) using the Pearson chi-squared test. The independent *t*-test was then used to examine the possible associations between gender and age, gender and age of onset, therapy and age, and therapy and age of onset; these were presented as mean ± standard deviation (SD). The therapeutic characteristics of the participants were also evaluated using the frequency test, and the level of statistical significance was set at *p* < 0.05.

## 3 Results

### 3.1 Gender differences in clinical, sociodemographic, and EEG characteristics of the participants

The 940 patients comprised 445 (47.4%) males and 495 (53.6%) females, who were diagnosed and treated over the period of 5 years between January 2017 and December 2021. The average duration of follow-up was 2 years, and the mean age of onset at diagnosis was 33.29 ± 21.02 years. There was a significantly higher mean age of onset in males than females (35.19 ± 21.10 vs. 31.58 ± 20.82 years, p = 0.009). A higher percentage of females had tertiary education (211 (51.0%) vs. 203 (49.0%)) than males, while a higher percentage of males had postgraduate education (53 (56.4%) vs. 41 (43.6%)) over females, and these trends were statistically significant (p = 0.005) (see Table 1).

**TABLE 1:** Gender differences in the clinical and sociodemographic characteristics of the participants.

Variables	Categories	Male (N = 445)	Female (N = 495)	Statistics	*p*-value
AGE N (%)				2.625	0.269
	18–35	218 (46.1)	255 (53.9)		
	36–64	166 (46.8)	189 (53.2)		
	>65	61 (54.5)	51 (45.5)		
AGE (mean ± SD)		40.44 ± 19.24	38.07 ± 18.34	t = 1.932	0.054
MARITAL STATUS N (%)				10.698	0.058
	Never married	187 (48.8)	196 (51.2)		
	Currently married	254 (47.1)	285 (52.9)		
	Separated	1 (50.0)	1 (50.0)		
	Widow/widower	0 (0.0)	11 (100)		
	Cohabiting	1 (50.0)	1 (50.0)		
	Divorced	2 (66.7)	1 (33.3)		
FORMAL EDUCATION N (%)				15.060	0.005^a^
	None	17 (37.8)	28 (62.2)		
	Primary	8 (21.6)	29 (78.4)		
	Secondary	164 (46.9)	186 (53.1)		
	Tertiary	203 (49.0)	211 (51.0)		
	Postgraduate	53 (56.4)	41 (43.6)		
AGE OF ONSET (mean ± SD)		35.19 ± 21.10	31.58 ± 20.82	1.372	0.009^a^
EPILEPSY TYPE				1.591	0.661
	Focal onset	54 (48.6)	57 (51.4)		
	Generalized onset	316 (46.9)	358 (53.1)		
	Combined onset	55 (45.8)	65 (54.2)		
	Unclassified	20 (57.1)	15 (42.9)		
DURATION OF SEIZURES				15.968	<0.001^a^
	<2 Years	288 (53.4)	205 (41.6)		
	>2 Years	317 (70.9)	130 (29.1)		
SEIZURE FREQUENCY				2.343	0.126
	<6 Months	404 (48.2)	434 (51.8)		
	>6 Months	41 (40.2)	61 (59.8)		
SEIZURE REMISSION				4.523	0.104
	<6 Months	222 (50.9)	214 (49.1)		
	6 Months to 2 years	121 (45.5)	145 (54.5)		
	>2 Years	102 (42.9)	136 (57.1)		
ETIOLOGY				4.863	0.433
	Structural	114 (49.4)	117 (23.6)		
	Metabolic	54 (46.6)	62 (12.5)		
	Immune	1 (100)	0 (0.0)		
	Genetic	3 (25.0)	9 (1.8)		
	Infectious	4 (0.9)	8 (1.6)		

^a^ Statistically significant, SD- standard deviation.

### 3.2 Therapeutic characteristics among the participants

Of the 940 PWEs recruited, 604 (64.3%) were on ASMs (monotherapy: 504 (83.4%); polytherapy: 100 (16.6%)) while 336 (35.7%) were not on any ASMs. With regard to the type of medications, 477 (79.0%), 79 (13.0%), 21 (3.2%), 19 (3.4%), and 8 (1.4%) participants were on carbamazepine, levetiracetam, valproate, phenytoin, and phenobarbiturate, respectively (see Table 2).

**TABLE 2:** Therapeutic characteristics among the participants.

Variable A	Frequency	Percentage N (%)
No use of ASMs	336	35.6
Use of ASMs	604	64.4
Total	940	100
Variable B		
Carbamazepine	477	79
Levetiracetam	79	13
Valproate	21	3.2
Phenytoin	19	3.4
Phenobarbiturate	8	1.4
Total	604	100

^a^ Statistically significant, ASM, Antiseizure medication, Variable A- Frequency of ASM use, Variable B- Prescription pattern of ASM.

### 3.3 Comparing clinical and sociodemographic characteristics among patients on monotherapy and polytherapy

The mean age of onset in PWEs on monotherapy was 34.47 ± 21.80 vs. 25.39 ± 19.78 years (*p* < 0.001) compared to those receiving polytherapy. Furthermore, there were significant differences in monotherapy and polytherapy with regard to age (p = 0.002), marital status (p = 0.006), and duration of seizures (p = 0.012) (see Table 3).

**TABLE 3:** Comparison of clinical and sociodemographic characteristics among patients receiving monotherapy and polytherapy.

Variables	Categories	Monotherapy = 504 N (%)	Polytherapy = 100 N (%)	X^2^ value	p-value
AGE				12.530	0.002^a^
	18–35	237 (73.2)	66 (21.8)		
	36–64	194 (87.8)	27 (12.2)		
	>65	73 (91.2)	7 (8.8)		
AGE (mean ± SD)		40.92 ± 19.40	33.61 ± 16.51	t = 3.523	<0.001^a^
GENDER				0.476	0.490
	Male	233 (46.2)	50 (50.0)		
	Female	271 (53.8)	50 (50.0)		
MARITAL STATUS				16.409	0.006^a^
	Never married	187 (77.9)	53 (22.1)		
	Currently married	305 (87.1)	45 (12.9)		
	Separated	0 (0.0)	1 (100)		
	Widow/widower	9 (100)	0 (0.0)		
	Cohabiting	1 (100)	0 (0.0)		
	Divorced	2 (66.7)	1 (33.3)		
AGE OF ONSET (mean ± SD)		34.47 ± 21.80	25.39 ± 19.78	7.214	<0.001^a^
FORMAL EDUCATION				9.321	0.054
	None	26 (92.9)	2 (7.1)		
	Primary	23 (76.7)	7 (23.3)		
	Secondary	201 (85.9)	234 (14.1)		
	Tertiary	209 (79.5)	263 (20.5)		
	Postgraduate	45 (91.8)	49 (8.2)		
DURATION OF SEIZURES				6.374	0.012^a^
	<2 Years	251 (87.5)	36 (12.5)		
	>2 Years	253 (79.8)	64 (20.2)		
SEIZURE FREQUENCY				0.036	0.850
	<6 Months	437 (83.6)	86 (16.4)		
	>6 Months	67 (82.7)	14 (17.3)		
SEIZURE REMISSION				1.036	0.596
	<6 Months	226 (81.9)	50 (18.1)		
	6 Months to 2 years	153 (84.1)	29 (15.9)		
	>2 Years	125 (85.6)	21 (14.4)		
TYPE OF EPILEPSY				6.077	0.108
	Focal onset	53 (81.5)	12 (18.5)		
	Generalized onset	369 (84.8)	66 (15.2)		
	Combined onset	60 (75.0)	20 (25.0)		
	Unclassified	22 (91.7)	2 (8.3)		
ETIOLOGY				7.411	0.116
	Structural	144 (87.8)	20 (12.2)		
	Metabolic	54 (93.1)	4 (6.9)		
	Immune	1 (100)	0 (0.0)		
	Genetic	5 (62.5)	3 (37.5)		
	Infectious	5 (100)	0 (0.0)		

^a^ Statistically Significant, SD, standard deviation.

## 4 Discussion

### 4.1 Sociodemographic characteristics

In this study, there is a preponderance of the female sex (males/females = 0.89), which is similar to the findings in previous reports from studies in Nigeria, Rwanda, and Tanzania but inconsistent with findings from studies from other SSA countries (Osuntokun et al., 1987; Hirose, 2014; Assadeck et al., 2019). The findings from the majority of the participants aged 18–35 years are in tandem with demographics. Notably, 79% of Nigerians are below the age of 35 or 40 (NPC, 2006). Furthermore, infections of the central nervous system (CNS), especially parasitic infections like neurocysticercosis; trauma, birth asphyxia, and other perinatal morbidities; ineffective immunization strategies against infectious diseases; as well as poverty and lower standards of living are possible additional reasons for the increased incidence of epilepsy among adolescents and younger adults (Paul et al., 2012; Singh et al., 2022). Previously, a meta-analysis study showed bimodal age grouping among the PWEs, which is similar to the findings of the current study that there were more epilepsy patients in the age groups of 18–35 and 36–64 years (Singh et al., 2022). Gender diversity has been observed in epilepsy research conducted within communities in Nigeria, highlighting variations in the occurrences of epilepsy between males and females. Studies by several researchers have reported higher prevalences of epilepsy among females in certain communities, while other investigations have shown higher prevalences among males (Osuntokun and Odeku, 1970; Owolabi et al., 2020; Watila et al., 2021). The exact reasons for these gender differences in epilepsy rates are not fully understood but could be influenced by various biological, hormonal, genetic, and sociocultural factors. In addition, females with epilepsy face unique challenges throughout their lifetimes owing to the effects of seizures and ASMs on sexual development, the menstrual cycle, fertility, pregnancy, menopause, and bone health caused by hormonal fluctuations (Harden and Pennell, 2013; Menon et al., 2019; Reddy, 2022).

### 4.2 Treatment gaps

The medication characteristics of the participants in this study showed that 336 (35.7%) of the 940 PWEs recruited were not on medications, which raises questions about the therapeutic and diagnostic gaps in epilepsy in SSA countries. Additionally, the scarcity of essential medications contributes to this problem, leaving many patients without access to vital treatments. The reported size of the epilepsy TG varies widely from 23% to 100%, though there has been a progressive decrease in SSA countries overall (Komolafe et al., 2012; Ogunrin et al., 2013; Watila et al., 2021). In this study, nearly a third of the participants did not receive ASM prescriptions; an immediate explanation for this cannot be attributed to the serious TG, which may be worsened by other determinants of non-adherence to medication. The TGs documented in previous studies range from 23% to as high as 90% (Assadeck et al., 2019). Delayed presentation and seeking of alternatives to medicines, such as healing homes, herbalists, and other spiritual mission houses, have been ascribed to the high rates of TGs observed in low- and middle-income countries. There are dramatic global disparities in the care of epilepsy between high- and low-income countries as well as between rural and urban settings (Owolabi et al., 2020; Singh et al., 2022). There is a substantial epilepsy TG in Nigeria, as indicated by Owolabi et al. on this issue. In SSA countries, the scarcity of trained healthcare professionals, especially in rural areas, has widened the burden of TGs in epilepsy. However, a recent estimate of the TGs in SSA countries indicated that the overall prevalence was about 68.5% (95% confidence interval (CI): 59.5%–77.5%). Studies conducted in various parts of Nigeria have shown epilepsy TGs ranging from 12.2% to 96%, which are comparable to the average value 68.5% obtained in general for SSA countries.

### 4.3 Patterns of ASMs

Of the 940 PWEs in the cohort, 477 (79.0% of those on ASMs) were on CBZ, and 79 (13.0% of those on ASMs) were on levetiracetam. This finding aligns with the reports from other studies that showed over 70%–97.5% use of CBZ among the PWE cohorts in SSA countries (Nwani et al., 2013; Sanya et al., 2013; Assadeck et al., 2019; Owolabi et al., 2020). Comparatively, the prescription of levetiracetam, a new-generation ASM, in this study was high compared to similar studies in SSA countries. A study by Assadeck et al. (2019) showed that there was limited access to newer ASMs and that only 45 (72.6%) people were on CBZ, 15 (24.2%) were on levetiracetam, and 15 (3.2%) were on phenobarbital among the epileptic patients. In a study carried out in Taiwan on a cohort of 118,937 PWEs, valproic acid was the most prescribed ASM (Schmidt and Gram, 1995). However, this is different from the findings in our study, which showed that CBZ was the most prescribed medication. A possible reason for this could be the preponderance of female subjects in this study and the usual practice of avoiding the use of sodium valproate in females of childbearing age because of its effects on the reproductive system.

In terms of drug therapy, the majority of PWEs on ASMs were on monotherapy (504 persons or 83.4%) while the rest were on polytherapy (100 persons or 16.6%). This is in line with various studies that have shown that monotherapy is preferable in the treatment of PWEs. However, if monotherapy fails, then polytherapy is recommended, especially in cases of drug-resistant epilepsy (DRE) (Thomas et al., 2005; Yoo and Panov, 2019; Verrotti et al., 2020a; Verrotti et al., 2020b). In the present study, the use of monotherapy was common among the PWEs, while polytherapy was frequently used among those of younger age and those with seizure durations exceeding 2 years. This further underscores the use of polytherapy among PWEs with prolonged or refractory epilepsy.

Monotherapy has been promoted as an ideal epilepsy treatment by various guidelines because of the lower side effects, limited drug interactions, better compliance, lower cost, and improved seizure control compared to polytherapy (Schmidt and Gram, 1995; Guberman, 1998). The National Institute for Health and Care Excellence (NICE) guidelines state that seizure type, age, gender, comorbidities, adverse drug reactions (ADRs), potential drug–drug interactions, availability, and affordability must be considered when choosing ASMs. The combination of ASMs may also result in pharmacodynamic synergistic effects that may not only increase efficacy but also more usage of ASMs (Verrotti et al., 2020a).

### 4.4 Strengths and limitations of the study

This study is limited to only epilepsy patients with no control; thus, the characteristics of the epilepsy cohorts cannot be compared to those of the controls. The retrospective nature of this study is another major limitation. However, to avoid recall bias, we performed a robust review of the medical records of the participant case notes obtained from the respective health departments of three hospitals. Even with these limitations, this study provides further insights into the prescription patterns of ASMs and the burden of TGs among the PWEs in SSA countries.

## 5 Conclusion

The majority of participants receiving ASMs were on monotherapy, with CBZ being the most frequently prescribed medication, which conforms with the guidelines of ASM use that favors monotherapy over polytherapy in achieving seizure control and improving the quality of life. Furthermore, about a third of the PWEs had TGs, which significantly identifies TG as one of the factors mitigating optimal epilepsy treatment among the PWEs, underscoring the need for a multifaceted strategy to address such gaps. To mitigate such TGs, healthcare providers should prioritize eliminating stigma and increasing public awareness of the condition, improving accessibility to healthcare services, and ensuring affordability and availability of ASMs.

## Data Availability

The raw data supporting the conclusions of this article will be made available by the authors without undue reservation.
